# Current Understanding of the Role of Adenosine Receptors in Cancer

**DOI:** 10.3390/molecules29153501

**Published:** 2024-07-26

**Authors:** Katharigatta Narayanaswamy Venugopala, Michela Buccioni

**Affiliations:** 1 Department of Pharmaceutical Sciences, College of Clinical Pharmacy, King Faisal University, Al-Ahsa 31982, Saudi Arabia; 2 Department of Biotechnology and Food Science, Faculty of Applied Sciences, Durban University of Technology, Durban 4001, South Africa; 3 School of Pharmacy, Medicinal Chemistry Unit, ChIP, University of Camerino, Via Madonna delle Carceri, 62032 Camerino, Italy; michela.buccioni@unicam.it

**Keywords:** adenosine receptors, A_1_, A_2A_, A_2B_, and A_3_ adenosine receptors, cancer, tumor

## Abstract

Cancer, a complex array of diseases, involves the unbridled proliferation and dissemination of aberrant cells in the body, forming tumors that can infiltrate neighboring tissues and metastasize to distant sites. With over 200 types, each cancer has unique attributes, risks, and treatment avenues. Therapeutic options encompass surgery, chemotherapy, radiation therapy, hormone therapy, immunotherapy, targeted therapy, or a blend of these methods. Yet, these treatments face challenges like late-stage diagnoses, tumor diversity, severe side effects, drug resistance, targeted drug delivery hurdles, and cost barriers. Despite these hurdles, advancements in cancer research, encompassing biology, genetics, and treatment, have enhanced early detection methods, treatment options, and survival rates. Adenosine receptors (ARs), including A_1_, A_2A_, A_2B_, and A_3_ subtypes, exhibit diverse roles in cancer progression, sometimes promoting or inhibiting tumor growth depending on the receptor subtype, cancer type, and tumor microenvironment. Research on AR ligands has revealed promising anticancer effects in lab studies and animal models, hinting at their potential as cancer therapeutics. Understanding the intricate signaling pathways and interactions of adenosine receptors in cancer is pivotal for crafting targeted therapies that optimize benefits while mitigating drawbacks. This review delves into each adenosine receptor subtype’s distinct roles and signaling pathways in cancer, shedding light on their potential as targets for improving cancer treatment outcomes.

## 1. Introduction

Cancer cells display numerous characteristics such as rapid proliferation, resistance to cell death, evasion of the immune system, sustained growth through limitless replication, formation of new blood vessels (neoangiogenesis), and the ability to spread to distant sites (metastasis). The continual persistence of malignant cells underscores the insufficiency of the host’s immune defenses in curbing tumor proliferation and metastasis. Malignant cells create an environment that suppresses the immune system, enabling their unrestricted proliferation and development [1,2,3,4]. Adenosine, classified as a purine nucleoside, can be either released from cells or generated externally through the degradation of ATP by two specific enzymes located on the cell surface: CD73 and CD39 [5,6]. Adenosine can inhibit several immune cells participating in antitumor activities and facilitate the formation of immunosuppressive cells like Tregs and Myeloid-derived suppressor cells (MDSCs) by interacting with adenosine receptors [7]. Studies have shown that both temporary and prolonged periods of low oxygen levels observed in solid tumors, known as hypoxia, lead to increased adenosine buildup in the tumor region [8]. Numerous researchers have documented the inhibition of antitumor responses by adenosine in both laboratory settings (in vitro) and living organisms (in vivo) through various studies [9,10,11,12]. Recent developments have demonstrated that blocking adenosine production can effectively halt tumor progression in living organisms (in vivo) [13]. The primary signaling pathway for A_1_ and A_3_ adenosine receptors (ARs) involves the inhibition of adenylyl cyclase (AC) and the resulting decrease in the cyclic adenosine monophosphate (cAMP) level [14,15]. Conversely, the activation of A_2A_ and A_2B_ adenosine receptors (ARs) induces the activation of adenylyl cyclase (AC), resulting in an increase in cAMP level [16,17]. Adenosine receptors (ARs) are extensively distributed across each part of the body, influencing a multitude of physiological and pathological processes across various organ systems, like the CNS, CVS, respiratory, urinary, and immune systems, among others.

Due to low oxygen levels in solid tumors, there is increased breakdown of adenine nucleotides, which raises adenosine levels in the tumor microenvironment. This excess adenosine is thought to boost the growth of new blood vessels (angiogenesis), which supports tumor growth. Adenosine is known to stimulate the production of vascular endothelial growth factor (VEGF) and encourages the movement and growth of endothelial cells. Adenosine also plays a role in slowing tumor growth, mainly by stopping cancer cells from dividing (cytostatic effect), rather than causing them to die (apoptosis). Interestingly, adenosine can directly hinder cancer cell growth at low concentrations (micromolar levels). However, it also suppresses the body’s immune response against tumors, which helps cancer cells evade destruction by the immune system [18,19].

As a result, ARs are considered promising targets for treatments in diverse medical contexts [20,21,22,23]. Several agonists, partial agonists, antagonists, and allosteric modulators targeting the body’s A_1_, A_2A_, A_2B_, and A_3_ adenosine receptors (ARs) are currently under investigation in clinical trials following their recent discovery, patenting, and study [24,25,26,27,28,29]. Furthermore, there is a suggestion that targeting adenosine receptors using specific antagonists is a potent and promising therapeutic strategy for cancer treatment [28,30]. This review is about clarifying the role that adenosine and adenosine receptors play in cancer development and investigating their therapeutic potential.

## 2. Adenosine Receptors

Adenosine exerts its effect by interacting with a set of four G-protein-coupled receptors (GPCRs) comprising A_1_, A_2A_, A_2B_, and A_3_ ARs [31]. These receptors vary in the types of G proteins they attract, the signaling pathways they initiate within target cells, and their affinity for adenosine. Specifically, the A_2B_ AR exhibits a lower affinity for adenosine in contrast to the high affinity observed in A_1_, A_2A_, and A_3_ ARs [29,32,33] (Table 1). Adenosine receptors have specific structures at the molecular level and work in a particular way to affect how cells respond to adenosine, influencing various processes in the body.

These seven transmembrane glycoprotein receptors are distributed throughout the body and interact with G proteins. A_1_ and A_3_ receptors and A_2A_ and A_2B_ receptors are roughly 49% and 59% similar, respectively. A_2B_ receptors have a reduced affinity for adenosine binding as compared to A_1_, A_2A_, and A_3_ AR. Stimulation of the A_1_ AR involves interaction with various pertussis toxin-sensitive G proteins (G_i_^1^, G_i_^2^, G_i_^3^, and G_0_), which can regulate AC, potassium channels, calcium channels, and phospholipase C (PLC). The A_2A_ AR increases AC activity by coupling with G_s_ or G_olf_ proteins, leading to elevated intracellular cAMP concentration upon stimulation. Via coupling with G_s_/G_q_ proteins, the A_2B_ AR enhances AC activity and stimulates PLC. Lastly, the A_3_ AR inhibits AC and stimulates PLC through interaction with G_i_ and G_q_ proteins, respectively [34]. Moreover, ARs can activate the MAPK (mitogen-activated protein kinase) signaling pathway in diverse cell types, which is essential for regulating cell proliferation and differentiation [35]. Multiple external signals contribute to these cascades, including input through GPCRs [36]. Activation of signaling cascades, resulting in elevated intracellular calcium levels, and activation of PLC and phospholipase D (PLD) contribute to cellular proliferation and apoptosis [37,38]. Activation of A_2A_ and A_2B_ receptors leads to an increase in AC activity via G_s_ proteins. G_s_ protein activation is the primary mechanism for A_2A_ ARs, and A_2B_ triggers PLC activity through the G_q_ protein. A_2B_ ARs primarily function through G_q_ activation, leading to an increase in inositol phosphate formation. A_2A_ adenosine receptors trigger two extra signaling pathways: heightened intracellular calcium levels and enhanced inositol phosphate production. This activation also stimulates protein kinase C through G_α15_ and G_α16_ proteins, which are impervious to pertussis toxin [39,40,41].

## 3. The Structure, Function, Mechanism of Action, Storage, Release, and Synthesis of Adenosine

Adenosine is continuously released in response to shifts in metabolic activity rather than being synthesized or stored in vesicles. It modulates neuronal activity before and after synaptic transmission and can subsequently be absorbed or metabolized [42,43].

### 3.1. Formation of Adenosine

The level of free adenosine is influenced by energy expenditure both within and outside the cell. During metabolic stress, which can arise from activities like muscle and nerve function or pathophysiological states like hypoxia and ischemia, the extracellular concentration of adenosine significantly rises [44,45]. The rise in adenosine concentrations has been linked to the activation of a self-regulating loop that safeguards organs from injury in response to stress [42]. Four distinct 5′-nucleotidase activities within mammalian tissues possess unique biochemical and molecular properties. Among these, ecto-5′-nucleotidase (e-N) is the enzymatic source for breaking down AMP into adenosine extracellularly. Earlier literature reviews indicate that adenosine production, both intracellularly and extracellularly, may also rely on the specific neuronal preparations utilized and, in some cases, on the technique employed for adenosine release [46,47].

#### 3.1.1. Extracellular Formation

**Figure 1:**
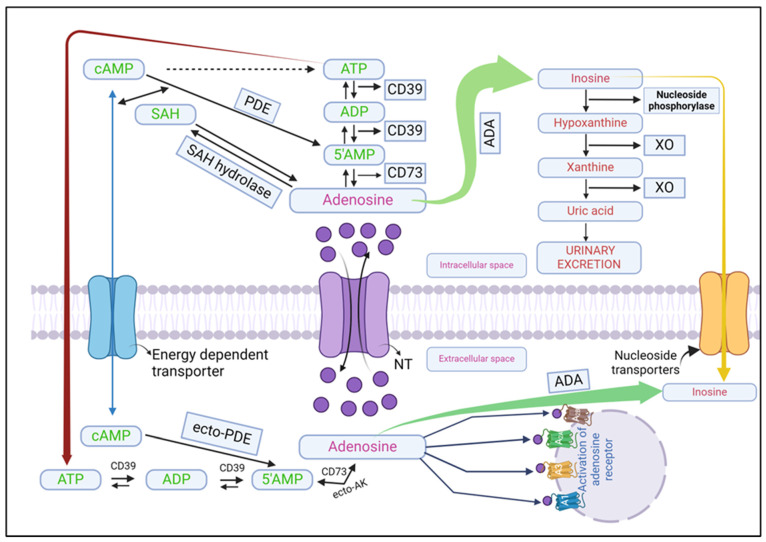
Synthesis, storage, release, function, and mechanism of action of adenosine. ADA (adenosine deaminase), ADP (adenosine diphosphate), ATP (adenosine triphosphate), cAMP (cyclic adenosine monophosphate), 5′AMP (5′ adenosine monophosphate), CD39 (ectonucleoside triphosphate diphosphohydrolase-1), CD73 (ecto-5′-nucleotidase), PDE (phosphodiesterase), SAH (*S*-adenosyl-homocysteine).

Two main processes induce extracellular adenosine generation: the first is the extracellular hydrolysis of adenine nucleotides or the external transport of intracellularly produced adenosine; the second is the extracellular breakdown of cAMP (Figure 1). Despite its involvement in numerous metabolic pathways, adenosine concentration within the intracellular space never reaches zero. Under basal conditions, the extracellular levels of adenosine have been observed to be restricted [48,49].

During stressful conditions, adenosine is predominantly generated in elevated levels through the dephosphorylation of AMP, cyclic adenosine diphosphate (ADP), and cyclic adenosine triphosphate (ATP) facilitated by two hydrolyzing enzymes: ecto-5′-nucleotidase (CD73) and ectonucleoside triphosphate diphosphohydrolase (CD39) [50]. Furthermore, ecto-phosphodiesterase (ecto-PDE), an extracellular enzyme that converts cAMP to AMP, can further promote adenosine production through CD73 [51,52].

In cancer, especially within the tumor microenvironment (TME), adenosine production is typically increased rather than decreased. This occurs because solid tumors often have areas with low oxygen levels (hypoxia); this hypoxic environment triggers cells in the tumor to increase the breakdown of ATP, which is a source of adenosine [53]. ATP is broken down into adenosine monophosphate (AMP) and then further into adenosine, which stimulate cells to break down ATP into adenosine [54].

Key enzymes involved in this process are CD39 and CD73. CD39 converts ATP into AMP, and CD73 further converts AMP into adenosine. These enzymes are upregulated in response to hypoxia and other signals within tumor cells, immune cells, and other TME cells [55]. Hypoxia also activates transcription factors known as hypoxia-inducible factors (HIFs), particularly HIF-1α, which regulate the expression of CD39 and CD73 genes. This ensures efficient adenosine production under low oxygen conditions [56]. Adenosine exerts its effects by binding to specific receptors (A_1_, A_2A_, A_2B_, A_3_) on cell surfaces, influencing processes that support tumor growth, suppress the immune response, and promote angiogenesis [57]. In summary, increased adenosine production in cancer and the TME, driven by hypoxia-induced upregulation of CD39 and CD73 enzymes, plays a crucial role in promoting tumor survival and growth through adenosine receptor signaling. Targeting these pathways could offer potential avenues for therapies aimed at counteracting adenosine-mediated tumor-promoting effects [58,59].

#### 3.1.2. Intracellular Formation

Within healthy cells, AMP undergoes hydrolysis by endo-5′-nucleotidase, and *S*-adenosyl-homocysteine (SAH) is hydrolyzed by *S*-adenosyl-homocysteine hydrolase (SAHH). These enzymatic processes result in the production of adenosine [48]. It is important to understand that SAH hydrolyzes reversibly to produce homocysteine and adenosine. Under thermodynamic equilibrium, adenosine and homocysteine are more likely to produce SAH, which raises SAH levels and prevents *S*-adenosylmethionine (SAMe) transmethylation. Therefore, transmethylation is facilitated by decreased adenosine concentrations, primarily regulated by adenosine kinase (ADK) [60].

As a result, adenosine production and elimination can be aided by SAHH [61,62,63]. The trans-methylation process, which involves the hydrolysis of SAH by SAH hydrolase to produce 1-homocysteine and adenosine, is an alternate mechanism for producing intracellular adenosine. In normal conditions, the amount of adenosine generated by this route is only one-third of that found in hypoxic cardiac situations [44].

### 3.2. Adenosine Release

In contrast to the vascular release of neurotransmitters triggered by electrical impulses, the release of adenosine is regulated through a nucleoside transporter mechanism. This led to the characterization and classification of two primary classes of transporters in 1980: equilibrative nucleoside transporters (ENTs) and concentrative nucleoside transporters (CNTs) [64]. After it is made, adenosine gets moved into cells through CNTs and ENTs.

Purine (adenosine) and pyrimidine nucleosides are transported across cell membranes by ENTs, which are passive, bidirectional nucleoside transporters. Through enhanced diffusion, these transporters assist in controlling the amount of adenosine in the extracellular area. They assist in maintaining a balanced concentration of adenosine across cellular membranes by using passive transport pathways for adenosine transfer, which eliminates the need for ATP or ionic gradients [65,66,67,68].

### 3.3. Adenosine Metabolism

Adenosine is rapidly transported to erythrocytes and vascular endothelial cells through synthetic and metabolic pathways, where it is catabolized (Figure 1). Within cells, adenosine is biotransformed into SAH, AMP, and inosine through hydrolysis, phosphorylation, and deamination, with the help of SAHH, adenosine kinase (ADK), and adenosine deaminase (ADA), respectively. ADK predominantly governs adenosine metabolism under normal physiological conditions, but ADA becomes more active in pathological conditions and facilitates adenosine clearance. Extracellular adenosine is cleared through ecto-ADA and is transported inwardly via ENTs (Figure 1) [21,69,70,71].

## 4. The Significance of Adenosine Receptors in Cancer

Adenosine, which accumulates in hypoxic areas of solid tumors, has been identified as capable of disrupting the ability of cytolytic effector cells from the immune system to recognize tumor cells [8,72]. Using lymphokine-activated killer (LAK) cells in adoptive immunotherapy has shown promise in treating malignancies that are resistant to standard therapies. Nevertheless, colon adenocarcinomas often exhibit limited responsiveness to LAK therapy, potentially due to tumor-induced immunosuppression. According to research, colon adenocarcinoma cells release a material distinct from prostaglandins (PGS) or TGF-β (transforming growth factor-beta) to halt the generation of anti-CD3-activated killer cells [11]. Adenosine has been proposed as a potential inhibitor of the solid cancer microenvironment’s cytotoxic T cell activation [10,73]. In fact, adenosine was found to inhibit adhesion by as much as 60% when it comes to the attachment of anti-CD3-activated killer (AK) lymphocytes generated from murine spleens to syngeneic MCA-38 colon cancer cells. Instead of the MCA-38 targets, AK cells were the subject of this preventive action, and the potency profile of agonists suggested that the adhesion inhibition may be due to the A_3_ AR subtype. The suggested concept is that immunosuppression resulting from tissue hypoxia could significantly contribute to the resistance of colorectal and other solid tumors to immunotherapy. Additionally, studies have demonstrated that adenosine significantly prevents the initiation of mouse cytotoxic T cells [74]. An A_3_ AR antagonist was shown to reverse the blocking effect of adenosine on the proliferation of AK-T cells, suggesting that adenosine activates A_3_ ARs to impede AK-T cell induction. In cancer patients, tumor-associated adenosine may prevent the formation of tumor-reactive T lymphocytes by a similar mechanism. Consequently, adenosine may play a part in the local immunosuppressive environment of solid tumors, as indicated by the suppression of T-killer cell function. According to later research, adenosine partially inhibits the interaction between tumor cells and T lymphocytes by interfering with the function of integrin α4β7, which acts as the primary cell adhesion molecule that makes it easier for T cells to adhere to syngeneic MCA-38 cells of adenocarcinoma [12]. 

Due to the typically inadequate oxygen supply, solid tumors often exist under hypoxic conditions. The breakdown of adenine nucleotides is facilitated by hypoxia, which raises the level of adenosine inside the tumor [75]. Adenosine is reported to be significantly more abundant in the extracellular fluid of solid tumors [8,76,77]. 

The release of adenosine after hypoxic conditions contributes to the augmentation of angiogenesis, thereby promoting tumor progression. Adenosine seems to stimulate the synthesis of vascular endothelial growth factors (VEGFs) and the proliferation of vascular endothelial cells, although there are conflicting findings regarding its impact on the secretion of angiogenic factors [78,79]. Adenosine’s stimulative effects on the synthesis of DNA [66] and endothelial cell migration contribute to its angiogenesis-promoting properties [67].

## 5. Molecular Signaling of A_1_ Adenosine Receptor and Its Role in Cancer 

**Figure 2:**
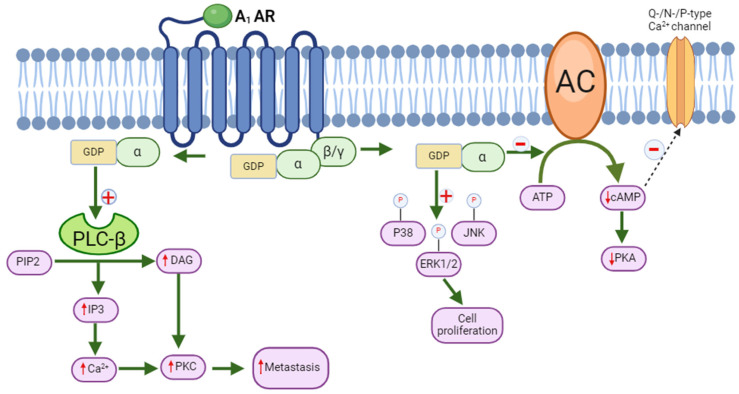
Molecular signaling of A_1A_ adenosine receptors. ATP (adenosine triphosphate), cAMP (cyclic adenosine monophosphate), DAG (diacylglycerol), ERK1/2 (extracellular signal-regulated kinases), GDP (guanosine diphosphate), IP3 (inositol trisphosphate), JNK (c-Jun N-terminal kinases), PKA (protein kinase A), PKC (protein kinase C), PIP2 (phosphatidylinositol 4,5-bisphosphate).

Stimulation of the G_i_-protein-coupled A_1_ adenosine receptor (A_1_ AR) triggers the suppression of AC, thereby causing a reduction in cAMP production [80]. Consequently, this reduction in cAMP levels leads to decreased phosphorylation of cAMP-dependent protein kinase A (PKA) and cAMP-responsive element-binding protein 1 (CREB-1) [81]. The A_1_ AR possesses the capacity to stimulate phospholipase C (PLC)-β, resulting in an elevated amount of diacylglycerol (DAG) and inositol trisphosphate (IP3). As a result, this raises calcium (Ca^2+^) concentrations inside the cell, which in turn triggers the activation of additional binding proteins and/or calcium-dependent protein kinase C (PKC) [21,81,82]. In neurons and cardiac tissue, activation of the A_1_ AR results in the opening of K^+^ (potassium) channels and the blocking of Q-type, P-type, and N-type calcium (Ca^2+^) channels (Figure 2) [83,84]. Moreover, A_1_ AR activation has been associated with the phosphorylation of mitogen-activated protein kinases (MAPKs) such as p38, ERK1/2, and JNK, and it also induces cell proliferation (Figure 2) [80].

Numerous studies have proposed the involvement of AC, cAMP, and cAMP response element-binding protein (CREB) in carcinogenesis and tumorigenesis [85,86,87,88,89]. Elevated expression of AC-3, observed in human gastric cancer cell lines and tissues, has been linked to the advancement of cancer. Additionally, heightened levels of cAMP, phosphorylated CREB, matrix metalloproteinase 2 (MMP2), and matrix metalloproteinase 9 (MMP9) were noted in HEK293 cells that overexpressed AC-3 (transfected with pAcGFP-ADCY3) [89]. Silencing of AC-3 has been found to inhibit tumorigenesis and cell proliferation. Additionally, AC-2 was found as a marker for an adverse result in colon cancer [88]. These results are consistent with the A_1_ AR agonists’ suppression of Sertoli-like TM4 cell proliferation and glioblastoma formation in the presence of cells called microglia [90,91]. Additionally, studies have shown that adenosine reduces microglial proliferation when an A_1_ AR agonist is present, whereas the absence of an A_1_ AR results in an increased density of microglia surrounding the tumor [91].

Adenosine receptors, particularly the A1AR, modulate tumor growth and immune responses within the tumor microenvironment (TME) by interacting with immune cells like microglia in brain tumors [92].

In cancer, particularly in the brain, low oxygen levels (hypoxia) in the TME produce a rise in adenosine levels. This increase in adenosine can activate the A_1_ AR in microglia. When the A_1_ AR is activated, microglia lose the ability to mount a powerful immune response against cancer cells. Adenosine’s inhibition of immune surveillance via the A_1_ AR promotes tumor growth and dissemination.

The A_1_ AR and other adenosine receptors also play a role in angiogenesis, the process by which new blood vessels are formed to supply nutrition and oxygen to tumors. Adenosine can cause the production of growth factors such as vascular endothelial growth factor (VEGF), which promotes the development of these blood vessels [93]. In terms of treatment, targeting adenosine receptors such as the A_1_ AR may improve the immune system’s ability to combat tumors. It may be possible to improve the immune system’s recognition and attack of cancer cells by blocking or altering how the A_1_ AR functions. This method could supplement current cancer treatments and enhance patient outcomes, particularly in tumors where microglia are influenced by adenosine signaling in the TME. Nevertheless, adenosine can stimulate microglial proliferation through cooperative interactions between A_1_ and A_2_ ARs [94]. A different investigation showed that an A_1_ AR antagonist prevented adenosine-induced apoptosis, whereas an A_1_ AR agonist caused human colonic cancer (CW2) cells to die. These results suggest that the A_1_ AR is involved in the tumor-suppressive properties of adenosine [95]. The overexpression of the A_1_ AR in malignancies may result from increased adenosine levels in the tumor microenvironment, which may be caused by hypoxia or oxygen scarcity [96,97]. The tumorigenic effects of the A_1_ AR were confirmed through the down-regulation of the A_1_ AR in mammary gland [97] and renal cell cancer [98] using RNA interference and an A_1_ AR antagonist, DPCPX, respectively. In addition to its ability to induce apoptosis in breast cancer cells, A_1_ AR siRNA impeded tumor growth and led to a halt in the cell cycle at the G2-phase /M-phase while reducing the cell population in the S-phase [97,99].

## 6. The Molecular Signaling Pathways Associated with A2 Adenosine Receptors and Their Involvement in Cancer

### 6.1. Molecular Signaling of Immunotherapy

**Figure 3:**
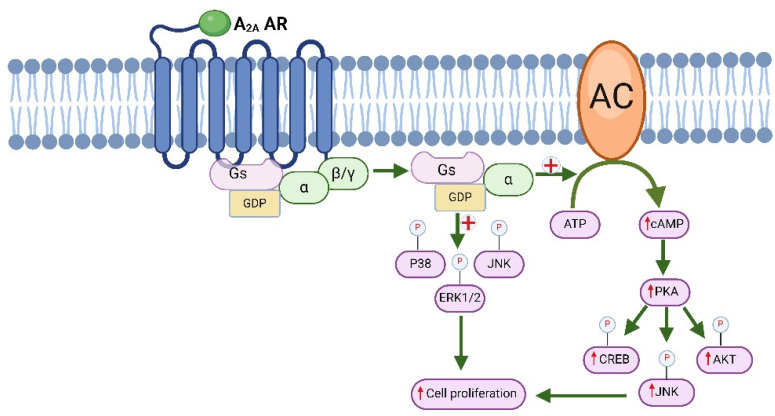
Molecular signaling of A_2A_ adenosine receptors. AKT (*protein-c*hinasi B), ATP (adenosine triphosphate), cAMP (cyclic adenosine monophosphate), CREB (cAMP response element-binding protein), DAG (diacylglycerol), ERK1/2 (extracellular signal-regulated kinases), GDP (guanosine diphosphate), IP3 (inositol trisphosphate), JNK (c-Jun N-terminal kinases), PKA (protein kinase A), PKC (protein kinase C), PIP2 (phosphatidylinositol 4,5-bisphosphate).

Stimulation of A_2A_ adenosine receptors (A_2A_ AR), which are linked to G_s_ proteins, activates adenylate cyclase (AC) [100]. Additionally, after ischemia-reperfusion injury (IRI), adenosine levels in the brain rise, which activates A_2A_ ARs. This activation worsens neuronal damage by increasing ERK (extracellular signal-regulated kinase) signaling. This process promotes microglial activation, the production of TNFα by glial cells, the release of glutamate, and the expression of inducible nitric oxide synthase (iNOS) and triggers apoptosis [101]. Studies have shown that in rat tail arteries, A_2A_ ARs can regulate the release of norepinephrine by activating PKA and PKC (protein kinase C) (Figure 3) [40]. Significantly, A_2A_ ARs can regulate the signaling of MAPK. Activation of A_2A_ ARs is crucial in cancer cells, as it promotes proliferation through the stimulation of PLC, protein kinase C delta (PKC-δ), ERK, JNK, and protein kinase B (AKT) pathways [102,103].

The purine nucleoside adenosine exhibits a strong affinity for the A_2A_ AR, which demonstrates varying expression levels across different tissues. Generally, A_2A_ AR expression is primarily observed in blood platelets, leukocytes, spleen, thymus, and the brain, whereas its occurrence in blood vessels, lungs, and the heart is moderate [104,105]. However, the increase in A_2A_ adenosine receptors (A_2A_ ARs) is closely linked to cancer, because adenosine plays a crucial role in controlling different stages of cancer growth. This includes promoting the formation of new blood vessels (angiogenesis), increasing the multiplication of cancer cells, helping cancer cells evade detection by the immune system, and facilitating their spread to other parts of the body (metastasis) [106,107]. It is suggested that having too many A_2A_ ARs in the cancer microenvironment could encourage uncontrolled growth of cancer cells [7,108].

Conversely, as previously discussed, the overexpression of A_2A_ ARs also impacts the recognition of cancerous cells by immune cells, including cytosolic T cells [72,102,109]. Moreover, the angiogenic properties of A_2A_ ARs contribute to wound healing and facilitate the proliferation of breast cancer and melanoma cells [110,111,112]. The expression of CD73 promotes cancer cell metastasis through A_2A_ ARs stimulation, whereas studies demonstrate that metastasis is impaired in mice with genetic mutations affecting the Adora2a gene, which encodes the A2A receptor. Additionally, various investigations into the blockade of A_2A_ ARs have demonstrated the inhibition of tumor growth as well as metastasis [113,114]. Consequently, an A_2A_ AR inhibitor may lessen the malignant state and enhance survival.

### 6.2. Molecular Signaling of A_2B_ Adenosine Receptors and Their Role in Cancer

Like the A_2A_ AR subtype, the A_2B_ AR is also linked to G_s_ protein, which activates AC, leading to increased cAMP concentration, phosphorylation of PKA, and cAMP-dependent recruitment of various effectors such as exchange proteins (Epac) [17]. It has been demonstrated that A_2B_ AR stimulation activates Epac (Exchange Protein Activated by cAMP), which influences umbilical vascular endothelial cell proliferation and initiates early gene expression, both of which decrease the smooth muscle cell proliferation in human coronary arteries [115,116,117]. In contrast to the A_2A_ AR, the A_2B_ AR is additionally coupled to G_q_ protein, which activates PLC, resulting in calcium ion (Ca^2+^) mobilization and the modulation of ion channels via recruiting γ subunits. By activating the MAPK and PKB pathways, the A_2B_ AR can modify a variety of pathological processes in the central and peripheral nervous systems [118,119,120]. Recent studies have shown that activation of A_2B_ AR leads to a reduction in ERK1/2 (extracellular signal-regulated kinases 1 and 2), p38, and NF-κB (nuclear factor kappa-light-chain-enhancer of activated B cells) signaling triggered by RANKL, thus diminishing osteoclastogenesis formation into the bone [121]. Numerous studies have highlighted the involvement of A_2B_ AR signaling in neuroinflammation [122,123], inflammatory bowel disease [124], cardiac ischemic preconditioning [125], atherosclerosis development [126,127], and attenuation of cardiac fibrosis.

Since the A_2B_ AR is expressed in the brain, circulatory system, immune system, astrocytes, neurons, and endothelial cells, the specificity of adenosine towards this receptor is notably low. Numerous illnesses, such as cancer, vascular diseases, chronic as well as acute lung diseases, problems with the urinary system, diabetes, and renal issues, have been linked to overexpression of this receptor subtype [118]. The oncogenic function of A_2B_ AR has been demonstrated through decreased cancer cell apoptosis triggered by TNFα and chemotherapy in prostate cancer cells overexpressing the A_2B_ AR [128,129]. The development of tumor growth by A_2B_ ARs is facilitated through several processes. In addition to the most common process involving accumulation of cAMP, activation of A_2B_ ARs initiates phospholipase-C-β stimulation through the associated G_q_ protein, leading to the subsequent IP3-dependent activation of PKC or the mobilization of the second messenger, calcium [129].

**Figure 4:**
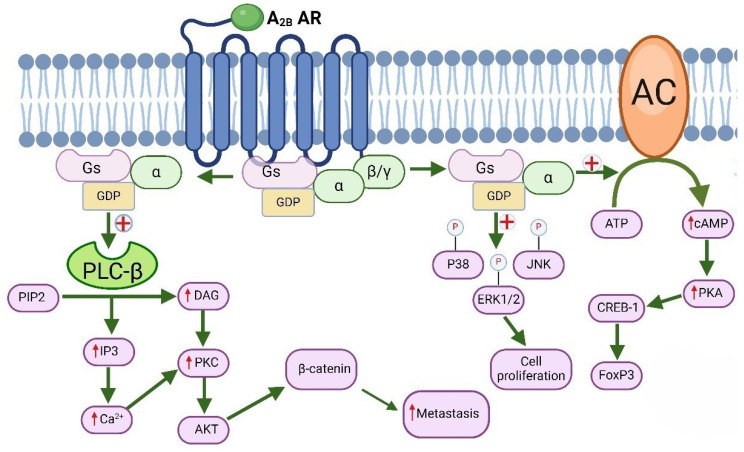
Molecular signaling of A_2B_ adenosine receptor and its role in cancer. AKT (*protein-c*hinasi B), ATP (adenosine triphosphate), cAMP (cyclic adenosine monophosphate), CREB (cAMP response element-binding protein), DAG (diacylglycerol), ERK1/2 (extracellular signal-regulated kinases), FoxP3 (forkhead box P3), GDP (guanosine diphosphate), IL-10 (interleukin 10), IP3 (inositol trisphosphate), JNK (c-Jun N-terminal kinases), PKA (protein kinase A), PKC (protein kinase C), PIP2 (phosphatidylinositol 4,5-bisphosphate), PLC-β (phospholipase C-β), TGF-β (Tumor Growth Factor-β).

Simultaneously, an elevated amount of cAMP triggers Epac, which blocks the T cell maturation and replication through the small GTPase Rap1. In response to stimulation of the T cell receptor (TCR), this attenuates MAPK signaling. Moreover, it is clear that the diffusion of cAMP from regulatory T cell gap junctions affects cancer cell signaling. A_2B_ AR activation promotes the early phases of metastatic function, such as cancer cell motility and migration [130]. It is interesting to note that A_2B_ ARs specifically activate MAPK signaling pathways to promote metastasis [131]. A_2B_ ARs have been linked to all three MAPK family members: JNK, stress-activated protein kinase p38, and ERK 1/2 (Figure 4) [132]. As cells start to disperse, A_2B_ ARs obstruct Rap1B’s location on the cell surface, beginning the first stages of cancer metastasis. Stimulation of A_2B_ ARs leads to the phosphorylation of Rap1B, a process that hinders its localization at the cell membrane [133]. Consequently, targeting the prevention of Rap1B phosphorylation could be a strategy to inhibit cell scattering during tumor metastasis.

Likewise, the presence of A_2B_ ARs on the surface of cancer cells is prompted by the pro-metastatic Fra-1 transcription factor. Inhibition of metastasis in Fra-1-expressing cells was observed upon antagonizing the overexpressed receptors with selective antagonists [134]. The expression of A_2B_ ARs on cancer cells suppresses the class-II trans-activator (CIITA), subsequently impairing MHC class-II transcription in cells stimulated with IFN-γ [135]. Hence, inhibiting the overexpression of A_2B_ ARs can restore the apoptotic potential and immune response of cancer cells, thereby enabling the control of cancer cell growth. Numerous studies in the literature confirm this, including evidence of the agonistic impact of BAY 60-6583 on A_2B_ ARs. This is demonstrated by its promotion of cancer cell proliferation and migration in mammary glands in vitro, as well as the induction of interleukin-10 production. Conversely, specific antagonism of A_2B_ ARs with ATL801 has been demonstrated to reduce the in vivo proliferation rate of 4T1 breast cancer and MB49 urinary bladder neoplasms [136].

## 7. Molecular Signaling of A_3_ Adenosine Receptor and Its Role in Cancer

**Figure 5:**
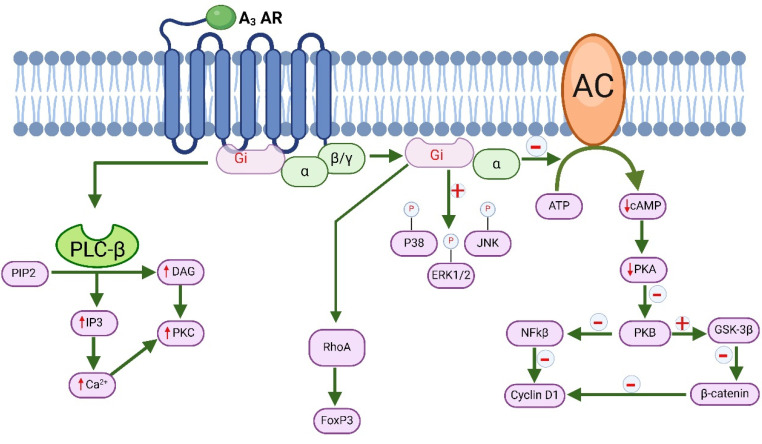
Molecular signaling of A_3_ adenosine receptor and its role in cancer. ATP (adenosine triphosphate), cAMP (cyclic adenosine monophosphate), DAG (diacylglycerol), ERK1/2 (extracellular signal-regulated kinases), FoxP3 (forkhead box P3), GSK-3β (glycogen synthase kinase-3β), IP3 (inositol trisphosphate), JNK (c-Jun N-terminal kinases), NFKβ (nuclear factor kappa-light-chain-enhancer of activated B cells), PKA (protein kinase A), PKB (protein kinase B), PKC (protein kinase C), PIP2 (phosphatidylinositol 4,5-bisphosphate), RhoA (ras homolog family member A).

The A_3_ AR subtype is linked to the G_i_ protein, leading to the inhibition of AC activity, resulting in decreased levels of cAMP. Additionally, at elevated agonist concentrations, the A_3_ AR can engage with G_q_ protein, thereby activating PLC and promoting the release of Ca^2+^ from intracellular stores [21]. Reduced levels of cAMP lead to the inhibition of PKA, resulting in the upregulation of glycogen synthase kinase-3β (GSK-3β). This decrease in cAMP also contributes to the reduction of β-catenin, cyclin D1, and c-Myc expression, while simultaneously diminishing the DNA-binding capability of NF-kB [137,138,139]. The neuroprotective and cardioprotective effects facilitated by the A_3_ ARs are regulated through distinct signaling pathways, which encompass G-protein RhoA and phospholipase D (PLD) [21]. The A_3_ adenosine receptor (A_3_ AR) has been found to activate ERK1/2 signaling pathways, which promote cell replication (shown in Figure 5) in different types of cells such as microglia, human fetal astrocytes, glioblastoma, melanoma, and others [117,140,141]. Interestingly, reduced ERK activation has been observed in prostate cancer, melanoma, and glioma cells. This reduced activation leads to decreased cell growth and less production of TNF-α [142,143]. A_3_ AR activation also affects how the JNK and p38 signaling pathways are modulated in a variety of cell types, including cancer cells like colon carcinoma [144].

**Figure 6:**
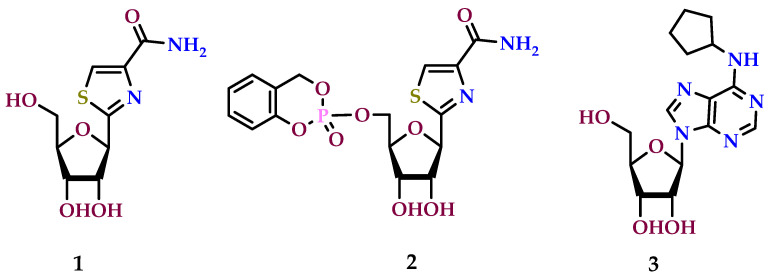
A_1_ adenosine receptor agonists with anticancer properties.

The A_3_ adenosine receptor is extensively expressed in various tissues, such as the CVS, CNS, respiratory system, and liver, albeit with varying levels of intensity. Additionally, it is present in various cells of the glial and immune cells [145]. Given its proven overexpression in a wide range of malignant cells and tissues, the A_3_ AR has excellent promise as a cancer biomarker and potential therapeutic target. The A_3_ AR has a beneficial and detrimental impact on cell proliferation and apoptosis in malignancies, most likely regulated by factors such as agonist concentration, cell type, contemporaneous adenosine receptor connections, and the tumor microenvironment [145,146,147]. Activation of the A_3_ adenosine receptor (A3 AR) in human glioblastoma cells triggers several pathways that contribute to increased invasiveness and the formation of new blood vessels (angiogenesis). Firstly, A3 AR activation stimulates the ERK 1/2 pathway, leading to the activation of proteins called extracellular signal-regulated kinases (ERKs). These ERKs increase the production and activity of MMP-9 (matrix metalloproteinase-9), an enzyme that breaks down the surrounding tissue matrix, allowing cancer cells to invade nearby tissues. Secondly, activation of the AKT/PKB pathway by A_3_ AR promotes the survival and growth of cancer cells, further supporting their invasive potential [144]. Additionally, adenosine acting through A3 ARs induces the expression of vascular endothelial growth factor (VEGF) by stabilizing a protein called hypoxia-inducible factor 1 (HIF-1). VEGF promotes the growth of new blood vessels, which helps tumors receive nutrients and oxygen, crucial for their growth. These processes highlight the significant role of A_3_ AR signaling in promoting aggressive behavior and the development of blood vessels in glioblastoma [25,130,148]. Increased levels of HIF-1 have been associated with higher cancer mortality rates, and inhibition of HIF-1 has been shown to decrease angiogenesis and tumor progression [149]. Down-regulation of A_3_ ARs using siRNA and the application of A_3_ AR antagonists have been shown to diminish chemoresistance to paclitaxel, consequently promoting natural cell death in glioblastoma cells [117]. On the contrary, stimulation of the A_3_ AR by the agonist 1-deoxy-1-[6-[[(3-iodophenyl)methyl]amino]-9*H*-purine-9-yl]-*N*-methyl-*β*-D-ribofuranuronamide (IBMECA) has been shown to regulate tumor growth-suppressive mechanisms in melanoma. These mechanisms involve receptor internalization, resynthesis, and externalization, ultimately leading to the deregulation of Wnt pathways [150]. Similar to A_1_ AR, activation of the A_3_ AR also down-regulates AC activity, leading to reduced levels of cAMP and PKA [63,151]. In the presence of adenosine, elevated levels of cAMP were detected in A_3_ AR knock-out mice [152]. Upon activation, the A_3_ AR inhibits PKA activities, thus preventing the subsequent GSK-3β phosphorylation or suppression. GSK-3β activation causes β-catenin to be phosphorylated or suppressed, resulting in lower levels of c-Myc and cyclin D1. This leads to reduced melanoma cell growth (Figure 6). In a separate study, activation of the A_3_ AR was discovered to inhibit PKA-mediated ERK 1/2 activation and subsequent NADPH oxidase activity in prostate cancer cells. This led to decreased cell proliferation and invasiveness [153]. On another note, the A_3_ AR has the potential to reduce cell proliferation by down-regulating the Akt/NF-κB signaling pathway. Moreover, the A_3_ AR agonist (CF102) shows protective effects against inflammation in the liver, as evidenced by reductions in serum levels of glutamic oxaloacetic transaminase and glutamic pyruvic transaminase, along with diminished concentration of NF-κB and TNF-α. These effects may be attributed to a decrease in the amounts of phosphorylated GSK-3β [154]. Additionally, CF102 demonstrated anticancer properties by promoting apoptosis by upregulating pro-apoptotic genes and activating caspases. Furthermore, A_3_ AR agonists have been shown to induce apoptosis in malignant mesothelioma [155] and leukemia cells [156]. The agonist 2-chloro-*N*-6-(3-iodobenzyl)-adenosine-5′-*N*-methyl-uronamide (Cl-IB-MECA) induced apoptosis and caused a halt in the cell cycle at the G_0-_/G_1_-phase. It reduced telomeric signals and suppressed metastasis in melanoma [157].

## 8. Adenosine Receptor Modulators: Potential Cancer Therapy 

Adenosine receptor modulators engage with adenosine receptors, impacting various physiological processes. These compounds exhibit potential in cancer treatment by targeting pathways vital to tumor growth, immune system avoidance, and inflammation. This approach presents opportunities to develop innovative therapies that may improve outcomes for cancer patients. Table 2 presents some of the important adenosine receptor modulators and their mechanism of action.

### 8.1. A_1_ AR Agonists and Antagonist

Tiazofurin (**1**) is recognized as an inosine monophosphate dehydrogenase (IMPDH) inhibitor, functioning as a C-nucleoside. Its clinical efficacy has been demonstrated in the context of anticancer treatment [158]. Tiazofurin undergoes conversion to tiazofurin adenine dinucleotide, effectively inhibiting the IMPDH enzyme. This inhibition leads to the suppression of nucleotide synthesis, thereby impeding the proliferation of tumor cells [99]. Nevertheless, the cyclosaligenyl-tiazofurin monophosphate (**2**), identified as a novel tiazofurin pronucleotide, has been discovered to act as a selective A_1_ AR agonist, displaying an affinity akin to tiazofurin [159]. Additionally, it showed in vitro efficacy against the human chronic myelogenous leukemia K-562 cell line [160]. The impact of A_1_ AR activity on the growth of glioblastoma cells was assessed using an organotypic brain slice model. In this model, glioblastoma cells were injected, allowing for the stimulation or inhibition of adenosine receptors [91]. Mice lacking the A_1_ adenosine receptor (A_1_ AR-deficient) and their wild-type littermate counterparts displayed distinct responses when injected with Gl261 glioblastoma tumor cells and subsequently treated with adenosine along with an A_1_ AR agonist, *N*^6^-cyclopentyladenosine (CPA), with CPA resulting in a notable reduction in tumor size. Notably, investigations revealed that adenosine or CPA (*N*^6^-cyclopentyladenosine) (**3**) did not influence tumor development in the brain slices obtained within A_1_ AR-deficient mice. These findings indicate that CPA (**3**) and adenosine exert their effects by specifically targeting A_1_ ARs on microglial cells, resulting in a reduction in tumor size [25,91]. Figure 6 provides structural representations of potential A_1_ AR agonists that exhibit anticancer action.

**Figure 7:**
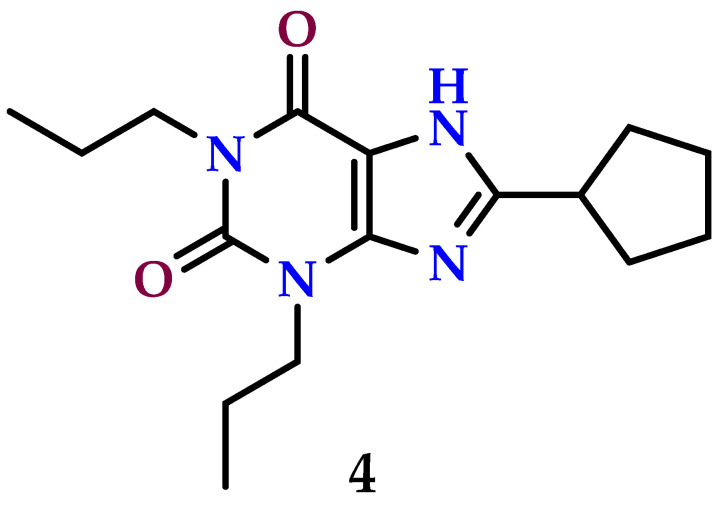
A_1_ receptor antagonists (1,3-dipropyl-8-cyclopentylxanthine, 4) with anticancer properties.

The A_1_ AR has a role in many malignancies, and its effects vary depending on the kind of cancer [161]. Its involvement in renal cell carcinoma (RCC) is currently being investigated using quantitative real-time PCR and Western blotting research on ACHN and 786-O cells [99]. Moreover, an anticancer study found that 1,3-dipropyl-8-cyclopentylxanthine (**4**) (Figure 7), which acts as an A_1_ AR antagonist, efficiently suppresses cancer of renal cell proliferation in vitro and inhibits tumor development in vivo. Compound **4** also demonstrated inhibition of RCC cell migration, whereas CPA, a selective A_1_ agonist, showed the ability to restore RCC cell migration. Furthermore, the xanthine derivative **4** enhanced cell death in ACHN and 786-O cells, causing a cell division stoppage in the S-phase cell cycle [99].

### 8.2. A_2A_ Receptor Agonists

**Figure 8:**
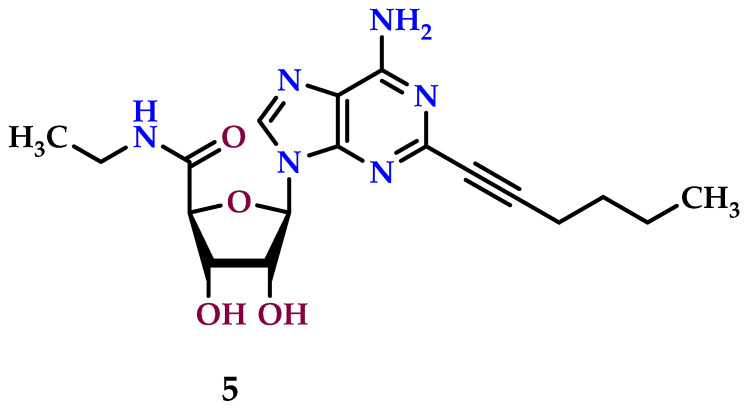
A_2A_ receptor agonist with anticancer properties.

The anticancer effects of A_2A_ receptor agonists were studied in A-375 cells employing a specifically selected A_2A_ agonist (2*S*,3*R*,5*R*), HENECA (2-hexynyl-NECA) (**5**) (Figure 8). Purine derivative compound **5** demonstrated mild but consistent cytotoxic and cell proliferation-inhibiting impacts on A-375 cell lines [162,163]. A_2A_ agonist compound **5** triggers cell death in a concentration-dependent manner, peaking in activity at approximately 100 nM. Nevertheless, its efficacy declines at higher concentrations [164].

### 8.3. A_2A_ Receptor Antagonists

**Figure 9:**
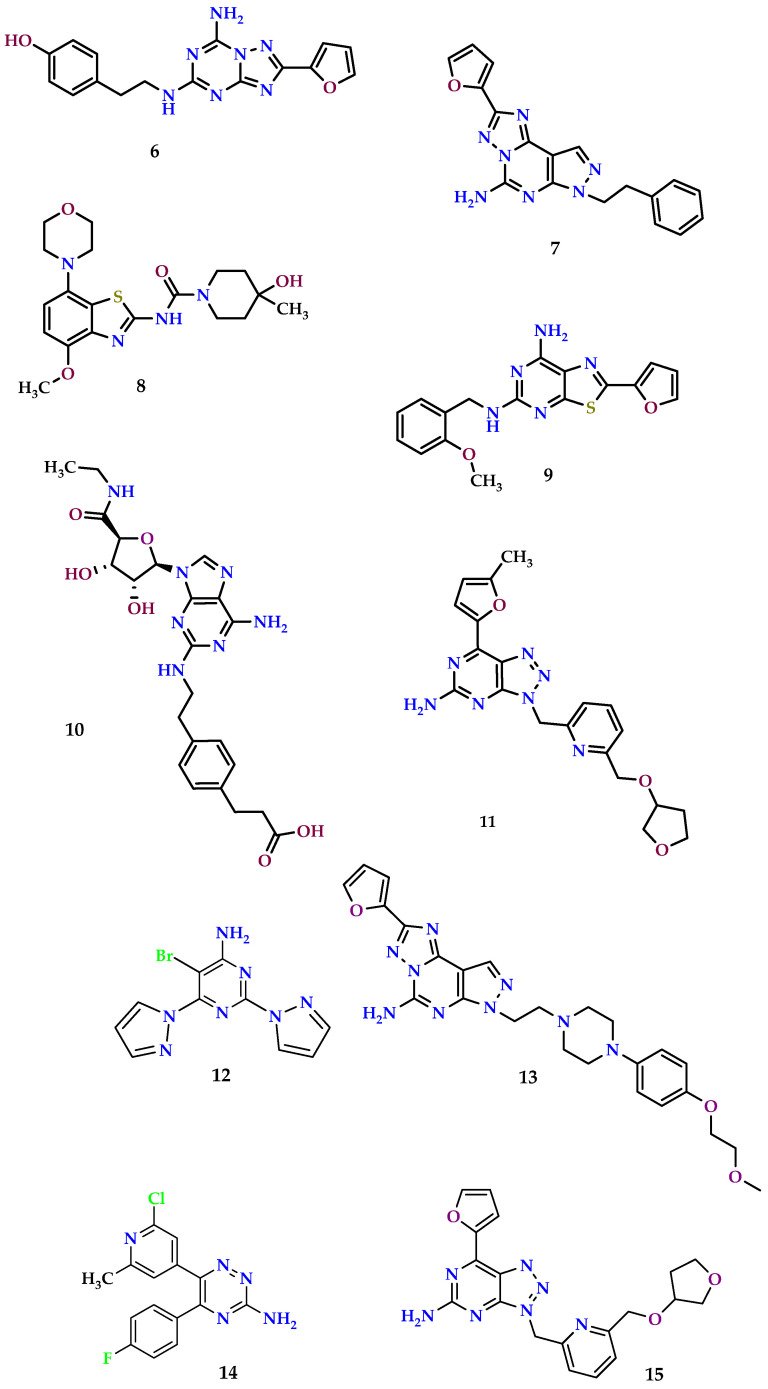
A_2A_ receptor antagonists with anticancer properties.

It is widely recognized that hypoxic solid tumors are associated with elevated levels of adenosine, resulting in decreased identification of cancer cells by cytolytic T lymphocytes, which are important in treating tumors [72,109]. The involvement of A_2A_ ARs in cancer treatment is attributed to their capacity to activate cytolytic T cells, consequently heightening the response against hypoxic tumor cells [165,166]. Studies have revealed that A_2A_ ARs promote angiogenesis and enhance the proliferation of melanoma and breast cancer cells, underscoring the significance of developing A_2A_ AR antagonists for tumor intervention [110,112]. Figure 9 illustrates examples of A_2A_ AR antagonists exhibiting anticancer properties.

The A_2A_ receptor antagonist ZM241385 (4-(2-((7-amino-2-(furan-2-yl)-[1,2,4]triazolo[1,5-a][1,3,5]triazin-5-yl)amino)ethyl)phenol)(**6**) enhances the suppression of tumor progression, eradication of cancer, and inhibition of neovascularization by antitumor T cells. In wild-type mice, the triazolo triazine derivative **6** notably postponed CL8-1 tumor growth, leading to the development of anti-CL8-1 CD8+ T cells. In adoptive immunotherapy models, co-administration of the antagonist (compound **6**) combined with anti-CD8+ T lymphocytes prevents the development of tumors, whereas administering it alone does not result in such an effect [167]. 

The effectiveness of the A_2A_ AR antagonist against tumors with compound **6**, in conjunction with an anti-CTLA4 monoclonal antibody, has been investigated in melanoma-bearing mice [159]. Mice treated solely with compound **6** exhibited notable inhibition of tumor development. Nevertheless, the combined therapy led to a notable postponement in tumor growth when compared to the control group or the administration of compound **6** individually. This effect is believed to be linked to heightened levels of tumor-infiltrating CD8^+^ T lymphocytes cells and decreased accumulation of TREGs (regulatory T cells) within the tumor tissue. Notably, mice treated solely with compound **6** demonstrated increased infiltration of CD8^+^ T lymphocyte cells and decreased levels of TREGs in cutaneous melanoma tissue [168].

A comparable study looked at the efficacy of the A_2A_ receptor antagonist SCH58261 (2-(furan-2-yl)-7-phenethyl-7*H*-pyrazolo[4,3-e][1,2,4]triazolo[1,5-c]pyrimidin-5-amine) (compound **7**) in preventing random metastasis in the 4T1.2 tumor model, representing a severe metastatic breast carcinoma cell line [159]. Studies indicated that inhibiting the A_2A_ AR with the triazolo pyrimidine derivative **7** substantially decreased the metastasis of B16F10 CD73+ tumors. Furthermore, therapy with the A_2A_ antagonist **7** significantly reduced metastasis of 4T1.2 tumors compared to the control group. These findings confirmed that the action of compound **7** was caused by blocking the A_2A_ AR. Moreover, A_2A_^−/−^ mice were significantly protected from the metastasis of B16-F10 CD73^+^ tumor cells [113]. Similarly, the A_2A_ antagonist **7** exhibited inefficacies in non-A_2A_^−/−^ mice, thereby confirming the drug specificity [113,169]. Compound **7** was additionally proven to extend living and decrease metastatic cancer stress in animal models of skin cancer and mammary cancer when provided concurrently with anti-PD-1 monoclonal antibody [170].

In a second study, the possible role of the benzothiazole derivative SYN115 (tozadenant) (compound **8**) and A_2A_ AR antagonist was investigated for its capacity to augment the anticancer effects of blocking-PD-1 monoclonal antibody. It was observed that A_2A_ AR blocking agent **8** significantly augmented the antitumor activity of the anti-PD-1 monoclonal antibody to a comparable extent as compound **7** [170].

The role of A_2A_ ARs in different human cancers, such as A-375 melanoma, A-549 lung cancer, and rat MRMT-1 breast cancer, has been extensively studied, with a particular focus on signaling pathways and the antagonistic effects of a novel A_2A_ AR antagonist (**9**) known as TP455 (2-(furan-2-yl)-*N*5-(2-methoxybenzyl)thiazolo[5,4-d]pyrimidine-5,7-diamine). Compound **9**, a thiazolo pyrimidine derivative, effectively blocked the cell growth induced by CGS21680 (compound **10**), a specific A_2A_ adenosine receptor agonist, in various cancer cell types. The agonist CGS21680 typically activates signaling pathways involving PLC, PKC-d, AKT, ERK1/2, and JNKs to stimulate cell proliferation. However, when compound **9** was present, it significantly reduced the agonist’s ability to activate these pathways, indicating that selective A_2A_AR antagonists like **9** could potentially be developed into new cancer treatments [102]. 

Research has shown that pyrimidine derivative **11** possesses potent immune activating and antitumor properties. Studies have shown that this compound effectively blocks the production of intracellular cAMP in cells exposed to 5′-*N*-ethylcarboxamidoadenosine (NECA), which is a stable analogue of adenosine. Additionally, activation of the A_2A_ AR suppresses the rapid phosphorylation of ERK triggered by T cell receptor activation [171]. This subsequently results in decreased production of IL-2 and IFN-α by stimulated T lymphocyte cells. However, inhibiting the A_2A_ AR with antagonist **11** reinstates cellular signaling pathways in T lymphocytes. The effectiveness of antagonist compound **11** for the A_2A_ AR was recently examined in MC38 and CT-26 syngeneic murine tumor models. Notably, in cured mice re-challenged with MC38 cells, new tumors failed to appear, indicating the induction of systemic antitumor immune memory by antagonist **11**. Moreover, in the MC38 model, the concurrent administration of compound **9** with an anti-PD-L1 monoclonal antibody synergistically suppressed tumor growth, leading to the elimination of tumors in nine out of ten treated mice. Similarly, in the CT26 model, the co-administration of compound **11** with anti-PD-1 resulted in enhanced inhibition of tumor growth and prolonged survival compared to the administration of antagonist **11** alone [172,173].

Adenosine accumulates at greater levels within the environment surrounding a tumor, where it activates the immune checkpoint mediated by the A_2A_ AR, thereby suppressing antitumor responses. Targeting this checkpoint holds promise for enhancing antitumor T cell responsiveness. As part of this effort, a novel A_2A_ AR antagonist, PBF-509 (5-bromo-2,6-di(1*H*-pyrazol-1-yl)pyrimidin-4-amine) (**12**), has been developed as a potential anticancer therapeutic specifically aimed at non-small-cell lung cancer [174]. Research indicates that the compound PBF-509 exhibits high specificity for the A_2A_ AR. In a mouse model, treatment with PBF-509 resulted in decreased lung metastasis compared to its control. Additionally, studies showed increased A_2A_ AR expression in CD4^+^ cells and varying expression levels in CD8^+^ cells in freshly obtained tumor-infiltrating lymphocytes from lung cancer patients. Research carried out in vitro demonstrated that human tumor-infiltrating lymphocytes exhibited enhanced responsiveness when treated with PBF-509 in conjunction with anti-PD-1 or anti-PD-L1 therapies [175]. These findings suggest that targeting A_2A_ AR inhibition could offer innovative immunotherapeutic strategies for non-small-cell lung cancer [176].

It is important to note that numerous clinical trials are currently underway for A2A AR antagonists in cancer therapy, either alone or in combination with various immunotherapies [177,178]. In addition, several molecules already studied for different pathologies are in clinical trials as antitumors, like Preladenant (compound **13**), AZD4365 (6-(2-chloro-6-methylpyridin-4-yl)-5-(4-fluorophenyl)-1,2,4-triazin-3-amine) (compound **14**) [179], and Ciforadenant (compound **15**) [180]. 

### 8.4. A_2B_ Receptor Antagonists

**Figure 10:**
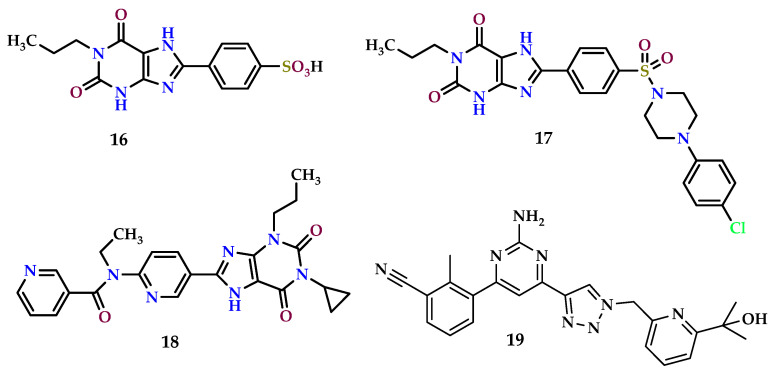
A_2B_ receptor antagonists with anticancer properties.

Activation of the A_2B_ AR is thought to promote the development of tumors through stimulation of angiogenic factor release from vascular smooth muscle, endothelial cells, and host immune cells [41,181]. On the flip side, blocking the A_2B_ AR results in heightened dendritic cell (DC) activation, which in turn leads to greater production of IFN-γ-inducible CXCL10 (C-X-C motif chemokine 10). This chemokine is responsible for activating lymphocytes and initiating an angiostatic response within tumors [182]. Several synthetic A_2B_ AR antagonists have demonstrated encouraging antitumor effects, as shown in Figure 10. 

Research has demonstrated that selective blockade of the A_2B_ AR using the antagonist PSB1115 (4-(2,6-dioxo-1-propyl-3,7-dihydropurin-8-yl)benzenesulfonic acid) (**16**) effectively delays melanoma growth. This delay is accomplished by blocking the buildup of myeloid-derived suppressor cells (MDSCs) within tumors and reinstating antitumor immune responses [183].

**Figure 11:**
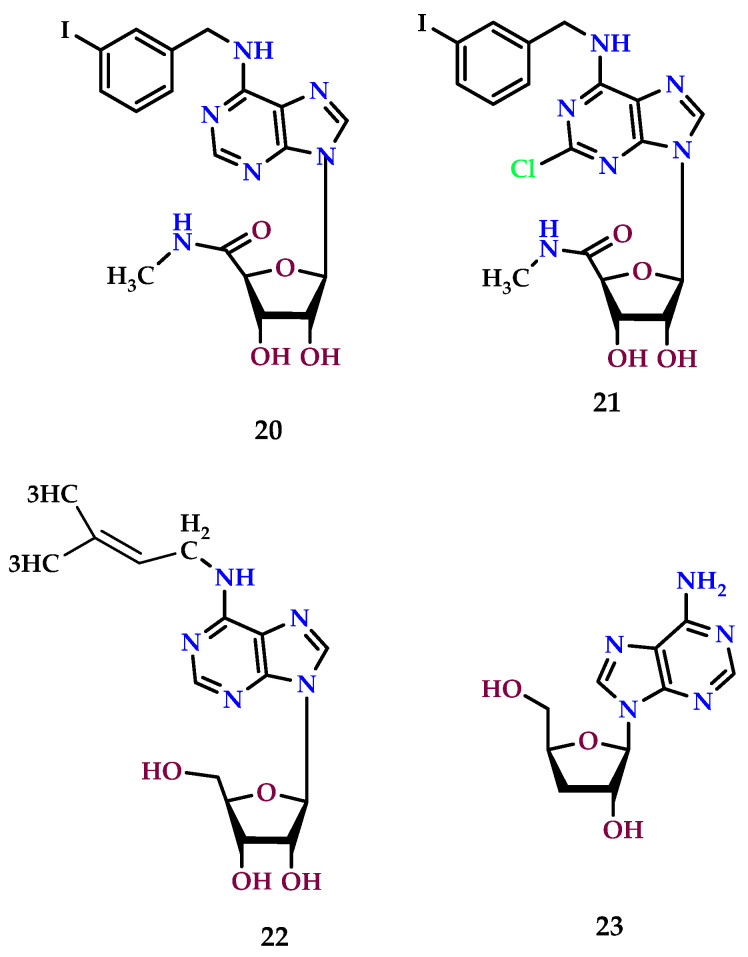
A_3_ receptor agonists have anticancer features.

Furthermore, research has demonstrated that inhibiting the A_2B_ AR with the selective antagonist **16** can reverse immune suppression in the tumor microenvironment, significantly slowing the progression of melanoma. The xanthine derivative **16** decreases the quantity of tumor-associated CD11b^+^Gr-1^+^ cells within melanoma cells as well as the levels of immune regulatory mediators like interleukin-10 (IL-10) and monocyte chemoattractant protein 1 (MCP-1). These effects are correlated with higher frequencies of natural killer T (NKT) cells and CD8-positive (CD8+) T cells that infiltrate tumors, as well as higher concentrations of cytokines that resemble T helper 1 (Th1). These results imply that the efficacy of the A_2B_ AR antagonist **16** depends on its ability to reduce the accumulation of myeloid-derived suppressor cells (MDSCs) that infiltrate tumors and to reestablish a potent antitumor T cell response. Moreover, it has been noted that xanthine derivative **16** blocking the A_2B_ AR significantly reduces the metastasis of B16F10 CD73^+^ tumors [170]. One of these xanthine derivatives is represented by PSB603 (8-[4-[4-(4-chlorophenzyl)piperazide-1-sulfonyl)phenyl]]-1-propylxanthine) (**17**) (Figure 11). This compound was evaluated in two different studies for its effect on melanoma cell lines and colorectal cancer cells [184,185,186].

High quantities of adenosine can accumulate in tumors and activate A_2B_ AR immune cells, impairing their ability to suppress tumor growth. However, A_2B_ AR antagonists can reverse this impact and restore immune cells’ anticancer activity. For example, the A_2B_ AR antagonist ATL801 (**18**) has been found to delay the growth of 4T1 breast and MB49 bladder tumors in syngeneic mice and prevent breast cancer cell metastasis. Intratumoral **18** administration can stimulate adaptive immune responses in a CXCR3-dependent manner, potentially through the indirect enhancement of dendritic cell (DC) activity, thereby counteracting the immunosuppressive effects of adenosine and inhibiting tumor growth. These results imply that focusing on the A_2B_ AR may provide a useful strategy for improving T cell activation and preventing angiogenesis in solid tumors [136]. 

Finally, it is worth highlighting that there are currently several antagonists in clinical trials for patients with various types of cancers. In particular, the dual-acting A_2A_/_A2B_ AB928 (**19**) exhibited excellent results in a Phase I clinical trial, and it is currently being evaluated in patients with ovarian cancer, non-small-cell lung cancer, and breast cancer [187,188].

### 8.5. A_3_ Receptor Agonists

The A_3_ AR coupled to G_i_ protein, known for its inhibitory regulative guanine nucleotide-binding properties, plays a pivotal role in mediating various beneficial effects such as anti-inflammatory, antineoplastic, and ischemia-reducing protective effects. Its overexpression in inflammatory and cancerous cells highlights its potential as a therapeutic target for cancer treatment [139]. The potential of A_3_ AR agonists with promising anticancer properties has been depicted in Figure 11.

The effect of A_3_ AR agonists on the proliferation of diverse tumor cells, such that by as piclidenoson (IB-MECA; **20**) and namodenoson (Cl-IB-MECA; **21**), is characterized by their remarkable affinity and selectivity for the A_3_ AR, even at minimal concentrations [189,190,191]. Significantly, these agonists exert distinct effects on tumor versus normal cell proliferation. 

Adenosine derivative **21**, in particular, increases the cytotoxic effect of chemotherapy, as demonstrated by colony formation assays and 1-(4,5-dimethylthiazol-2-yl)-3,5-diphenylformazan (MTT) assays. Compound **20** exhibits greater growth inhibition of HCT-116 human colon carcinoma cells when combined with 5-fluorouracil than when 5-fluorouracil is used alone [192]. On the other hand, at a concentration of 10 μM, the chloro-substituted adenosine derivative **18** inhibits the growth of human melanoma A375 cells by causing cell cycle arrest in the G_0-_/G_1_-phase [193].

Research has delved into the mechanisms underlying the impact of this ligand on estrogenic receptor α (ERα)-positive cells, revealing that derivative **21** swiftly down-regulates ERα at both mRNA and protein levels. This action inhibits proliferation or triggers apoptosis in various breast cancer cell types [193,194].

At low nanomolar concentrations, the synthetic A_3_ AR agonist **21** also inhibits the growth of HCT-116 human colon carcinoma cells, a response reversible by the selective A_3_ AR antagonist MRS1523 (**24**). This observation underscores the specificity of the agonist’s effects [195]. 

Similarly, the naturally occurring antitumor nucleoside N6-(2-isopentenyl) adenosine (**22**) exhibits high affinity and selectivity for A3 adenosine receptors (A3ARs). Moreover, the antiproliferative activity of adenosine derivative 22 can be completely inhibited by the selective A3 adenosine receptor antagonist, MRS1523 (24). This suggests that compound 20 exerts its antitumor effects at low concentrations by activating A3ARs [196].

It is noteworthy that cordycepin (3′-deoxyadenosine) (**23**), a natural compound found in Cordyceps sinensis, a parasitic fungus utilized in traditional Chinese medicine, also exerts growth inhibition on tumor cells through the A_3_ AR pathway [197]. This molecule demonstrates a significant suppressive effect on the growth of Lewis lung carcinoma LCC tumor cells and murine B16-BL6 melanoma at micromolar concentrations.

### 8.6. A_3_ Receptor Antagonist

A_3_ AR antagonists are commonly linked with anti-inflammatory properties and have also garnered attention for their potential as anticancer agents [198,199]. 

However, the role of A_3_ AR in tumors is controversial, and the antitumor effect of A_3_ AR ligands cannot be due exclusively to this receptor. Experimental evidence allowed hypothesizing that the effect is influenced by many aspects like the specific cell line, the concentration of the compound, and probably off-target effects within the cell. 

**Figure 12:**
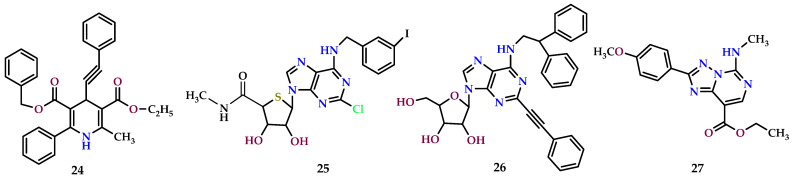
A_3_ receptor antagonist with anti-carcinogenic aspects.

The truncated thio-Cl-IB-MECA (**25**) (Figure 12) has been found to inhibit the growth of T24 human bladder cancer cells, primarily through its antagonistic effect on A_3_ AR. This inhibition manifests via sub-G_1_ cell cycle arrest, leading to both early and late apoptotic events [200]. A_3_ AR antagonists displayed a pronounced cytostatic activity on the prostate cancer cell line PC3, showing even more pronounced effects with respect to the reference agonist Cl-IB-MECA. In particular, *N^6^*-(2,2-diphenylethyl)-2-phenylethynylAdo (**26**) displayed the highest activity, proving to be a potential antitumor agent. The cytostatic effect exhibited by the A_3_ AR agonist (Cl-IB-MECA) and antagonists (**26** and other compounds of the series) highlight once more that the anticancer result is not due only to the contribution of A_3_ ARs, but other cellular mechanisms are implicated in the antitumor effects of these ligands [201].

A series of [1,2,4]-triazolo[1,5-c]pyrimidines also showed good antiproliferative effects on THP1 and HCT16 human tumor cell lines. In particular, the derivative **27** (Figure 12) proved to be particularly active against both cell lines, and in an attempt to clarify the antitumor effect of this compound, the authors evaluated its capacity to permeate the phospholipid bilayer of the cell membrane. The results of the Parallel Artificial Membrane Permeability Assay (PAMPA) showed that the compound can enter the cell and interact with potential target molecules, supporting the hypothesis of a parallel mechanism yet to be clarified [202]. 

A recent paper reports substitute adenosine derivatives simultaneously antagonizing the A_3_/A_2A_ AR having cancer immunotherapeutic activity. In fact, these not only exploit the antitumor signaling pathway activated through the inhibition of the A_3_ AR but also, as previously mentioned, inhibit the immune checkpoint mediated by the A_2A_ AR, suppressing aberrant immune responses [203]. 

## 9. Summary

Adenosine receptor (AR) signaling plays a pivotal role in cancer progression by influencing cell proliferation, tumor growth, angiogenesis, metastasis, and immune suppression. The elevated levels of adenosine within the tumor microenvironment activate all four AR subtypes (A_1_, A_2A_, A_2B_, and A_3_ ARs), making them potential targets for innovative cancer treatments. Because of their behavior in the tumor environment, A_2A_ adenosine receptors (A_2A_ ARs) activated by high adenosine levels and hypoxia decrease the immune response against tumors, potentially promoting tumor progression. It has been proposed that including A_2A_ AR blockers (antagonists) in cancer immunotherapy treatments could improve their efficacy by reversing immune suppression. Interestingly, the safety of A_2A_AR antagonists has already been tested in trials for Parkinson’s disease treatment. The role of A_2B_ adenosine receptors (A_2B_ ARs) in cancer is not fully understood: they can promote tumor growth by releasing substances that help create blood vessels, but they may also convey signals that inhibit tumor cell proliferation. As a result, it is uncertain whether medications that activate or inhibit A_2B_ ARs are more successful as anticancer therapy. Synthetic drugs that activate A_3_ ARs have been found to slow cell growth and promote cell death (apoptosis) in various cancer types, both in laboratory studies and animal models. Early clinical trials (Phase I/II) have shown that these drugs are safe and well tolerated in humans, implying that they could be useful for treating certain tumors like hepatocellular carcinoma (HCC), where they have shown significant effects in cancer cells. Blocking A_3_ ARs with specific drugs may prevent an increase in hypoxia-induced factors like HIF-1α, which promote blood vessel formation (angiogenesis) and cancer cell spread in the tumor environment. This approach could be a promising cancer treatment strategy. The use of adenosine receptor ligands has shown promising anticancer effects in both laboratory studies and animal models, leading to their consideration as potential drug candidates for cancer therapy. Specifically, activating the A_3_ AR or blocking A_1_, A_2A_, and A_2B_ receptors can potentially shift the cancer-promoting environment towards an anticancer state within the body. These ligands include nucleoside and nucleotide derivatives, adenosine analogues, and pyrimidine derivatives, among others. Though targeting adenosine receptors holds therapeutic promise, comprehensive research and clinical validation are necessary to elucidate their potential in cancer treatment fully. Studying the distinct roles of each adenosine receptor subtype is critical for developing targeted medicines that maximize benefits while minimizing drawbacks. Continued research into the signaling pathways, interactions with other molecules, and contextual effects of ARs in various cancer types is critical for progressing personalized cancer treatments. Finally, given the complexity of adenosine receptor signaling, modulation of these receptors has the potential to improve the outcomes of anticancer therapy in patients affected by this disease.

## Figures and Tables

**Table 1:** The molecular characteristics and mechanisms of action of adenosine receptors [27].

Receptors	Affinity for Adenosine (nM)	G Protein Coupling	Signaling System
A_1_ AR	1–10	G_i/o_	↓AC, ↑PLC, ↑PI3 kinase, ↑MAPK, ↑K^+^, Ca^2+^
A_2A_ AR	30	G_s_	↑AC, ↑MAPK
A_2B_ AR	1000	G_s_G_q/11_	↑AC, ↑PLC, ↑MAPK
A_3_ AR	100	G_s_G_q/11_	↓AC, ↑PLC, ↑PI3 kinase, ↑MAPK

**Table 2:** Adenosine receptor modulators and their mechanism of action.

Compounds	Target	Agonist/Antagonist	Mechanism of Action
Tiazofurin	A_1_ receptor	Agonist	Inosine monophosphate dehydrogenase (IMPDH) inhibitor
Cyclosaligenyl-tiazofurin monophosphate	A_1_ receptor	Agonist	Inhibition of inosine Monophosphate dehydrogenase (IMPDH)
*N*^6^-cyclopentyladenosine	A_1_ receptor	Agonist	Inhibition of adenylate cyclase
1,3-dipropyl-8-cyclopentylxanthine	A_1_ receptor	Antagonist	Binds and blocks the stimulation of A_1_ AR
HENECA (2-hexynyl-NECA)	A_2A_ receptor	Agonist	Stimulation of adenylate cyclase
ZM241385 (4-(2-((7-amino-2-(furan-2-yl)-[1,2,4]triazolo[1,5-a][1,3,5]triazin-5-yl)amino)ethyl)phenol)	A_2A_ receptor	Antagonist	Prevents A_2A_ receptor response
SCH58261 (2-(furan-2-yl)-7-phenethyl-7H-pyrazolo[4,3-e][1,2,4]triazolo[1,5-c]pyrimidin-5-amine)	A_2A_ receptor	Antagonist	Blocks A_2A_ receptor response
SYN115 (tozadenant)	A_2A_ receptor	Antagonist	Binds A_2A_ receptor and prevents the receptor from responding to its natural ligand (adenosine) or other agonists
TP455 (2-(furan-2-yl)-*N*5-(2-methoxybenzyl)thiazolo [5,4-d]pyrimidine-5,7-diamine)	A_2A_ receptor	Antagonist	Binds A_2A_ receptor and prevents the receptor from responding to its natural ligand (adenosine) or other agonists
CGS21680	A_2A_ receptor	Antagonist (in the presence of SYN115)	Blocks A_2A_ receptor
(NECA) 5′-*N*-ethylcarboxamidoadenosine	A_2A_ receptor	Antagonist	Blocks A_2A_ receptor
PBF-509 (5-bromo-2,6-di(1*H*-pyrazol-1-yl)pyrimidin-4-amine)	A_2A_ receptor	Antagonist	Binds A_2A_ receptor and prevents the receptor from responding to its natural ligand (adenosine) or other agonists
Preladenant	A_2A_ receptor	Antagonist	Blocks the adenosine A_2A_ receptor
AZD4365 (6-(2-chloro-6-methylpyridin-4-yl)-5-(4-fluorophenyl)-1,2,4-triazin-3-amine)	A_2A_ receptor	Antagonist	A_2A_ receptor inhibitor
Ciforadenant	A_2A_ receptor	Antagonist	Blocks A_2A_ receptor
PSB1115 (4-(2,6-dioxo-1-propyl-3,7-dihydropurin-8-yl)benzenesulfonic acid)	A_2B_ receptor	Antagonist	Blocks A_2B_ receptor
PSB603 (8-[4-[4-(4-chlorophenzyl)piperazide-1-sulfonyl)phenyl]]-1-propylxanthine)	A_2B_ receptor	Antagonist	A_2B_ receptor inhibitor
ATL801	A_2B_ receptor	Antagonist	A_2B_ receptor inhibitor
AB928	A_2B_ receptor	Antagonist	Adenosine A_2A_ and A_2B_ receptor blocker
Piclidenoson (IB-MECA)	A_3_ receptor	Agonist	Activation triggers intracellular signaling pathways mediated by G proteins, resulting in various downstream effects
(Cl-IB-MECA)	A_3_ receptor	Agonist	Activates the adenosine A_3_ receptor and triggers intracellular signaling pathways mediated by G proteins
N6-(2-isopentenyl)) adenosine	A_3_ receptor	Agonist	Activates A_3_ receptor
Cordycepin (3′-deoxyadenosine)	A_3_ receptor	Agonist	Activates A_3_ receptor
MRS1523	A_3_ receptor	Antagonist	Blocks the A_3_ receptor

## Data Availability

Not applicable.

## List of Abbreviations

A_1_ AR	A_1_ adenosine receptor
A_2A_AR	A_2A_ adenosine receptor
A_3_ AR	A_3_ adenosine receptor
AC	Adenylyl cyclase
ADP	Adenosine diphosphate
AMP	Adenosine monophosphate
AK	Adenosine kinase
AR	Adenosine receptors
ATP	Adenosine triphosphate
cAMP	Cyclic adenosine monophosphate
CD3-AK	CD3-activated killer
CIITA	Class-II trans-activator
CNS	Central nervous system
CNTs	Concentrative nucleoside transporters
CPA	Cyclopentyl adenosine
CREB	cAMP response element-binding protein
CREB-1	cAMP-responsive element-binding protein 1
CVS	Cardiovascular system
CXCL10	C-X-C motif chemokine 10
DAG	Diacylglycerol
DNA	Deoxyribonucleic acid
ecto-PDE	Ecto-phosphodiesterase
ENTs	Equilibrative nucleoside transporters
GPCRs	G-protein-coupled receptors
IP3	Inositol trisphosphate
iNOS	Inducible nitric oxide synthase
IRI	Ischemia-reperfusion injury
LAK	Lymphokine-activated killer
mAb	Monoclonal antibody
MAPKs	Mitogen-activated protein kinases
MDSCs	Myeloid-derived suppressor cells
MMP2	Matrix metalloproteinase 2
MMP9	Matrix metalloproteinase 9
PG_S_	Prostaglandins
PKA	Protein kinase A
PLC	Phospholipase C
PLD	Phospholipase D
RCC	Renal cell carcinoma
SAH	S-adenosyl-homocysteine
SAHH	S-adenosyl-homocysteine hydrolase
SAMe	S-adenosylmethionine
TGF-β	Transforming growth factor-beta
VEGFs	Vascular endothelial growth factors
